# ACE2 and COVID-19: using antihypertensive medications and pharmacogenetic considerations

**DOI:** 10.2217/pgs-2020-0048

**Published:** 2020-06-05

**Authors:** Eric M Snyder, Bruce D Johnson

**Affiliations:** 1 Geneticure, Inc., Four 3rd St. SW, Rochester, MN 55902, USA; 2 Mayo Clinic College of Medicine, 200 First St. SW, Rochester, MN 55902, USA

**Keywords:** ACE2, ACE-inhibition, angiotensin-receptor blockade, angiotensinogen, cardiac dysfunction, coronavirus, COVID-19, genomics, renin, respiratory failure

## Abstract

COVID-19 utilizes the ACE2 pathway as a means of infection. Early data on COVID-19 suggest heterogeneity in the severity of symptoms during transmission and infection ranging from no symptoms to death. The source of this heterogeneity is likely multifaceted and may have a genetic component. Demographic and clinical comorbidities associated with the severity of infection suggest that possible variants known to influence the renin–angiotensin–aldosterone (RAAS) system pathway (particularly those that influence ACE2) may contribute to the heterogenous infection response. ACE2 and Ang(1–7) (the product of ACE2) seem to have a protective effect on the pulmonary and cardiac systems. Hypertension medication modulation, may alter ACE2 and Ang(1–7), particularly in variants that have been shown to influence RAAS system function, which could be clinically useful in patients with COVID-19.

The novel coronavirus that emerged in 2019, like SARS outbreak of 2003, utilizes the ACE2 pathway as a means of cellular infection within the respiratory tract, among other organ systems throughout the body [1–3]. COVID19 utilizes a spike protein that mediates viral entry into the host cell with ACE2 as the host receptor [4]. The potential magnitude that COVID-19 has as a global pandemic has garnered unprecedented attention within and outside of the scientific community. While the long-term goals for treatment of COVID-19 include the development of a vaccine that can attenuate the effects of this coronavirus, along with the potential testing and utilization of antivirals that may be effective, there is evidence that the ACE2 pathway can be modulated with existing medications, particularly those along the renin–angiotensin–aldosterone (RAAS) pathway [5,6]. Care must be taken, however, because evidence from both human and animal models suggest differences in the importance of ACE2 (and the product of ACE2, Ang(1–7) on the susceptibility to the infection and the cardiorespiratory response to the infection may be opposite. Specifically, it is possible that low ACE2 levels may be associated with relatively low infection rates [1]. However, there is abundant evidence in models of acute lung injury (ALI) and acute respiratory distress syndrome (ARDS) that lower ACE2 and Ang(1–7) levels are associated with poorer prognosis [3,7,8].

The early data on COVID-19 demonstrate that there is extreme heterogeneity in the severity of symptoms with transmission and infection. Some individuals have demonstrated transmission with no to mild symptoms, some have moderate symptoms that resolve, and others have severe symptoms that result in respiratory failure and, in extreme cases, death. These early data have demonstrated that younger individuals have no to relatively mild symptoms, whereas older individuals, men and individuals with hypertension and diabetes have demonstrated more severe symptoms, on average [9–11]. Once infected, the common causes of severe morbidity and mortality include ARDS and cardiac dysfunction [10]. These points are extremely important in that low ACE2 levels may be important in minimizing risk of infection with transmission (young animals, including humans, demonstrate low ACE2 levels [particularly before puberty], male animals have demonstrated higher ACE2 levels, and both hypertension and diabetes are associated with high ACE2 levels) [12]. However, after infection with coronavirus, ACE2 and Ang(1–7) seem to have a protective effect on the pulmonary (reducing the severity of respiratory distress, pulmonary fibrosis, vascular leakage and pulmonary edema) and cardiac (reduction of cardiac injury, fibrosis, inhibition of cardiac hypertrophy and a reduction in the time of development of heart failure from cardiac hypertrophy in animal models) systems [3,7,9,13]. Using the early available data from COVID-19, that demonstrates comorbidities associated with high ACE2 levels are more likely to be infected, one can hypothesize that there are potential genetic variants that also influence the response to infection, given that common variants of renin, angiotensinogen, ACE and the Ang-II receptor have been shown to influence the function of the RAAS [14–18]. More recent data has also identified variants of *ACE2* that have important differences in minor allele frequency between populations, but the mechanistic importance of these has yet to be clearly defined [19]. Therefore, it is possible, given the known heterogenous response of COVID-19, along with the importance of ACE2 for infection, that variants along the RAAS pathway may be a reason for differential infection symptoms and infection severity.

Given the unique paradigm of how ACE2 influences infection transmission and then the cardiac and pulmonary response to the infection, it is possible that there are differences in the desired ACE2 and Ang(1–7) levels for minimization of the infection and, then, the attenuation of the cardiopulmonary effects of the infection once infection has occurred. Interestingly, common hypertension medication modulation, including use of renin inhibitors, ACE inhibitors, and Ang-II receptor blockers (ARBs) may play a role in modulation of ACE2, based on previous studies [20,21]. These same medications have been shown to have a differential blood pressure lowering response based on common genetic variants of the RAAS [22,23]. This potential to modulate the infection, and complications from the infection, is important because it would be ideal to minimize these two while more sweeping and dramatic treatments are being developed. It is also possible that ACE2 modulation in response to RAAS medication adjustments may differ according to known functional genetic variants within the RAAS that have been previously shown to impact this pathway.

## RAAS pathway & ACE2

There is evidence that the affinity of coronaviruses for ACE2 is important for viral infection [24,25]. ACE2 is a relatively newly discovered enzyme in the RAAS pathway. ACE2 is a homolog of the ACE but appears to be counteractive in nature to the function and activities of ACE and the product of ACE, Ang-II [5]. Within the RAAS pathway, renin cleaves angiotensinogen resulting in Ang-I. ACE then converts Ang-I to Ang-II, which is a primary drug target for patients with hypertension. Ang-II primarily binds to two types of receptors, the AT1 and AT2 receptors. ACE2 hydrolyzes Ang-I to make Ang(1–9) and hydrolyzes Ang-II to make Ang(1–7) that then binds to a GPCR MAS [6,26]. ACE, ACE2, Ang-II and AT1 receptor levels are high in the respiratory tract and lung, which seems to be the primary site of complications from the corona-type viruses [6]. It is thought that ACE2 activity may counteract the hypertensive effects of Ang-II in patients with high blood pressure, particularly early in hypertension, meaning that hypertension may increase endogenous ACE2 levels [5,6,27]. In addition to the lungs, ACE2 is expressed in the kidneys, nasal epithelium, vasculature, gastrointestinal system and pneumocytes [1,28]. Functional variants of renin, angiotensinogen, ACE and the AT1 receptor have been studied in detail and common variants have been shown to alter plasma levels, activity and the response to pharmacotherapy [22,23]. More recently, several common and functional genotypes of *ACE2* have been described, one of these seems particularly impactful on modulation of ACE2 levels [19,29,30]. Therefore, while it is likely that a portion of the differences in COVID-19 infection rates and severity are influenced by environment or traditional demographic variables known to influence ACE2 levels, the known heterogeneity of this infection may also have genetic associations.

## Evidence that ACE2 may be associated with COVID-19 infection

The early data from patients who have higher rates of infection and who have had more severe COVID-19 suggest that there are two primary common comorbidities: hypertension and diabetes [10]. In recent trials on COVID-19, these comorbidities comprised 23.7 and 30% (for hypertension) and 16.2% and 12% (for diabetes) of patients who develop more severe symptoms [11]. The increase in ACE2 activity in patients with hypertension has been demonstrated [31–33]. It is also possible that the administration of certain antihypertensive medications leads to an increase in ACE2 expression and/or activity [21,34]. In addition to hypertension, ACE2 levels have been shown to be elevated in animal models of both Type 1 and Type 2 diabetes [20,32,35,36]. Previous research has demonstrated that ACE2 expression (measured protein levels) and activity were higher in a db/db mouse model for diabetes and this same trial demonstrated that ACE activity is lower in these same mice, highlighting an important function of the in ACE/ACE2 ratio in health and disease [37].

In addition to hypertension and diabetes being common comorbidities for COVID-19, older individuals and males are more susceptible to the infection (and have demonstrated more severe cardiopulmonary complications from coronavirus), both of which are associated with differences in ACE2 levels. Early COVID-19 data, and data from SARS-CoV have shown a disproportionate influence on males, when compared with females [10]. ACE2 levels increase markedly in male sheep with age [38]. Specifically, ACE2 activity in sheep increases about 300% in males at one year while females had no change throughout development [38]. Age also influences ACE2 expression (mRNA and protein) in animal models and the sex differences in sheep do not emerge until post puberty [38]. Most studies demonstrate a decrease in ACE2 levels in older animals, and there may be a sex-dependent effect similar to animal models given the importance of estrogen on this pathway [39]. In additional work in rats, female rats had lower renal ACE2 activity compared with males and this difference was dependent on ovaries and ovarian hormones [40]. These data could explain why younger individuals seem to be able to transmit the virus, with lower levels of infection.

Using these comorbidity and demographic data available with early data on COVID-19 as a surrogate, using groups that have previously been shown to be associated with high ACE2 levels showing higher infection rates and severity of infection, it is possible that variation in ACE2 levels may alter the course of COVID-19. Given this, it is also possible that variants along the RAAS that are likely to influence ACE2 expression and/or activity may also influence the susceptibility to infection, severity of infection and potential complications, once infected.

## The importance of ACE2 & Ang(1-7) augmentations post diagnosis with coronavirus respiratory infection

**Figure 1.**
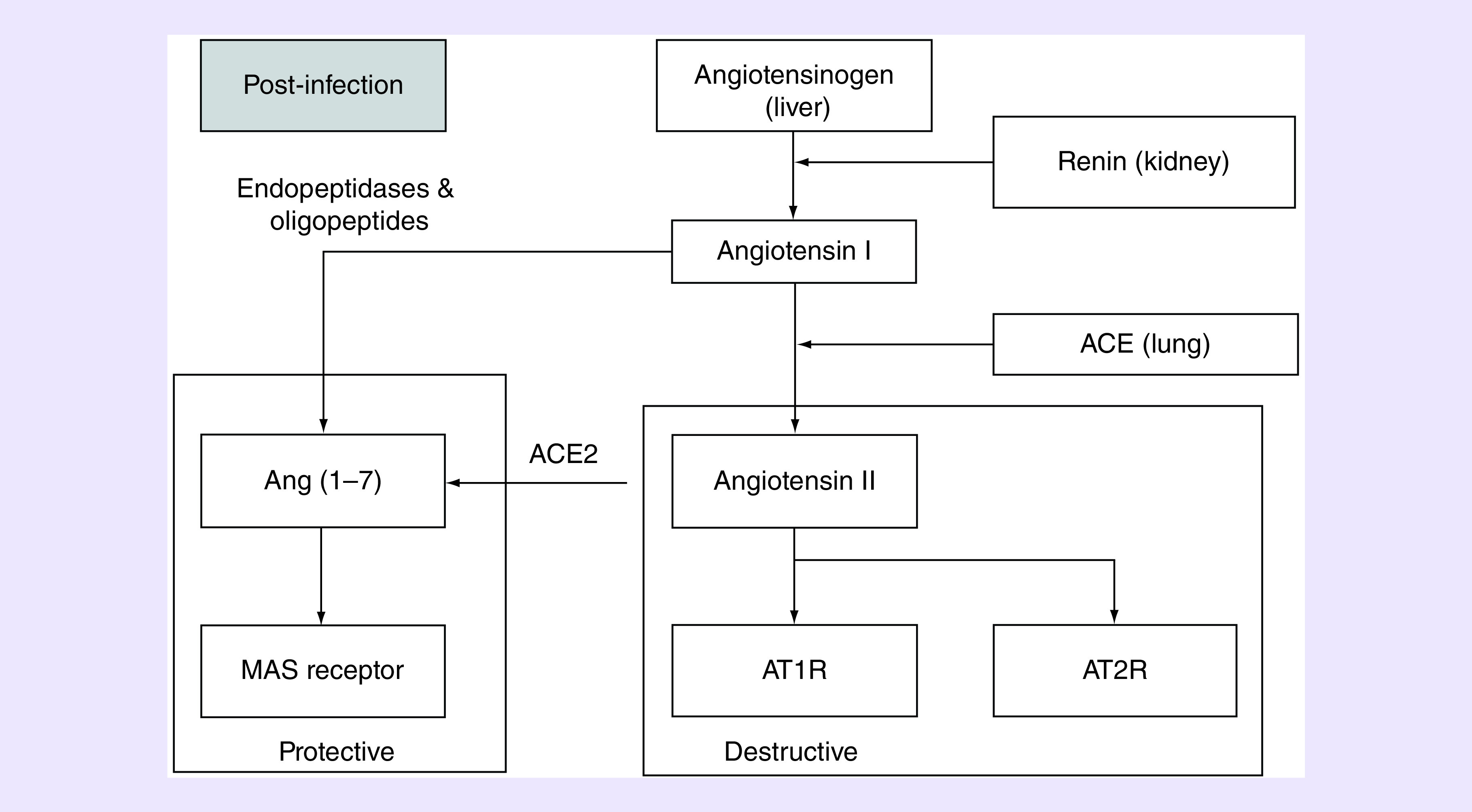
Schematic representation of ACE2 within the renin–angiotensin–aldosterone pathway. Once infected, it is likely that augmentations of ACE2 and Ang(1–7) will be protective for the cardiac and pulmonary systems. In contrast Ang-II and the Type 1 receptor of Ang-II have been shown to be destructive to the cardiac system and result in pulmonary leakage and fibrosis.

Interestingly, there is strong evidence that decreased ACE2 and Ang(1–7) levels are associated with poor prognosis in patients with respiratory dysfunction, including ALI and ARDS, similar to COVID-19 [3,8,41]. The ACE2/Ang(1–7) relationship with ACE/Ang-II is complex in that ACE2, and particularly Ang(1–7), have been shown to be primarily protective while ACE, and particularly Ang-II result in cardiopulmonary dysfunction (Figure 1). The primary cause of hospitalization in COVID-19 cases has been respiratory distress [10]. Also of interest, ACE2 and Ang(1–7) are cardioprotective, and a large number of patients that exhibit complications with COVID-19 demonstrate cardiac complications, including cardiomyopathy and heart failure [9]. It is possible that the protective mechanism of ACE2 and Ang(1–7) in the lungs and the heart are attenuated, with infection, and augmentation of this pathway may reduce the severity of the illness.

### Lung

ACE2 has been shown to be localized to the lungs, as has one of the angiotensin receptors, AT1, but not AT2. There is a clear and important interaction of ACE2 and AT1 on lung protection in models of disease, including those caused by coronaviruses [7,8,42]. Genetic knock-out of AT1a receptor expression markedly improved lung function in mice with a genetic knockout of this receptor (Agtr1a^-/-^ mice), confirming the function of Ang-II in lung health [5,43]. Additional work has shown that pharmacologic inhibition of the AT1 receptor attenuates the severity of acid-induced lung injury in ACE2 knockout mice and the loss of ACE2 expression in ALI leads to leaky pulmonary blood vessels through AT1a receptor stimulation [7]. ACE2 knockout mice developed more severe ALI in models that result in respiratory distress [41]. In models of acute ALI, acid-treated mice who have been genetically modified with a knockout for ACE2 have more lung elastase, when compared with wild-type mice, and this loss of ACE2 results in more pronounced deoxygenation, lung edema and increased inflammatory cell infiltration [7]. Additional work has demonstrated that ACE2 decreases fibroblast mitigation in pulmonary fibrosis [43]. Finally, injection of ACE2 seems to protect mice with ALI [7]. Previous research has demonstrated that the injection of SARS virus leads to an increase in Ang-II levels in lung tissue and exacerbates ALI in mice. Additionally, the viral spike from SARS seems to inhibit ACE2 that may result in increases levels of Ang-II. It is also possible that differences in ACE levels may influence pulmonary function and disease severity via an upstream (upstream of Ang-II) mechanism. An indirect influence of ACE on ACE2 is possible given that ACE levels will dictate the amount of Ang-II (for which ACE2 has a great affinity) and given the evidence that ACE-inhibition can increase ACE2 expression/activity in humans. There is one particular site of the *ACE* gene that has been studied in great detail, the presence (insertion, I) or absence (deletion, D) of a 287 base pair sequence of alu within intron 16 of the gene that encodes ACE. In fact, the D genotype of *ACE* is associated with ARDS susceptibility and outcome, where DD genotype was associated with ARDS [44], although this has not been demonstrated in all populations [45]. This same genotype of *ACE* is also associated with higher ACE activity, which would, theoretically, result in lower Ang-II levels. More recent data on COVID-19 has demonstrated important differences in race on infection rates and severity. Although this racial difference has been almost entirely attributed to the environment, there are known differences in minor allele frequency of several of the functional genes within the RAAS, which could also play a role [46–48]. It is also possible that other variants along the RAAS that could influence ACE2 levels (renin, angiotensinogen and the AT1a receptor) could alter endogenous ACE2 levels and be differentially affected by modulation using ACE-inhibitors or ARBs.

### Heart

Previous work in coronaviruses has demonstrated that a high percentage of patients with complications have cardiac dysfunction, including cardiomyopathy and cardiac injury. A recently published trial demonstrated that a greater number of patients hospitalized with COVID-19 demonstrate cardiac injury [9]. It is possible that this cardiac dysfunction following the viral infection, is also due to a temporary drop in ACE2 levels, due to ACE2 destruction as a result of the infection. Previous evidence indicates that the infection can result in ACE2 destruction, reduction in membrane-bound ACE2 and that ACE2 has a protective role in the heart [13,49]. The disruption of ACE2 accelerates cardiac hypertrophy and shortens the transition period to heart failure in an Ang-II model of heart failure [29]. In addition, pharmacologic inhibition of ACE2 exacerbates cardiac hypertrophy and circulating Ang(1–7) levels in transgenic rats are associated with reduced cardiac hypertrophy and fibrosis following isoproterenol infusion [50,51]. The attenuation of Ang-II can help with cardiac remodeling in that both ACE inhibition and the administration of ARBs increase ACE2 activity and/or expression.

It is possible that patients who become infected with COVID-19 lose some of the pulmonary and cardiac protective mechanisms of ACE2 and Ang(1–7), due to viral binding and the resultant destruction of ACE2 following infection [49]. It is likely that those who have less severe disease/infection/death rates are in groups associated with higher ACE2 activity after infection, either due to genetic differences, demographic variables or medication utilization. In addition, it is possible that modification of some common antihypertensive medications including ACE-inhibitors and ARBs may augment ACE2 post infection and protect the cardiopulmonary system, although this remains a hypothesis and, clearly, medication adjustments should never be made without the consultation with a patient's clinician [34].

## Modifications of ACE2 with medications that alter the RAAS pathway

ACE2 has a 400–500 fold lower affinity for Ang-I than for Ang-II [43]. Ang-II is also a primary target for pharmacotherapy, along with ACE, which converts Ang-I into Ang-II. It is, therefore, plausible that alterations in Ang-II and ACE expression and/or activity because of more versus less functional genetic variants along the RAAS pathway could, theoretically alter ACE2 activity. Further, since the pathway is also dependent on renin, it is possible that reductions in renin levels, through renin inhibition, may alter ACE2 expression and/or activity. In fact, previous work has demonstrated that the administration of an ACE-inhibitor or an ARB causes an increase in Ang(1–7) levels [34]. In addition, both ACE-inhibition and ARB administration increase the activity, and expression, of ACE2, which may be differential based on variants of ACE and Ang-II that have previously been shown to alter enzyme and receptor function, respectively [34].

Given this complex interaction along a pathway that is commonly treated with antihypertensive medications, it is possible that pharmacotherapy can target this pathway in established ways to attenuate ACE2 levels and reduce the likelihood for infections from COVID-19 and that augmentation of this same pathway, increasing ACE2 and Ang(1–7) may be beneficial, post infection. Specifically, ACE-inhibition and ARB administration may protect against cardiopulmonary dysfunction following COVID-19 infection. These considerations may be particularly impactful when considering genetic variants along this same pathway that have previously demonstrated meaningful functionality in humans.

## Conclusion

COVID-19 has rapid cause of dramatic morbidity and mortality worldwide. Many scientific teams are working toward long-term solutions that will attenuate/ablate COVID-19 but there may be potential to reduce transmission through reductions of ACE2 levels, and reduce cardiac and pulmonary complications, post infection, with the modulation of common medications along the RAAS pathway while more sweeping solutions are being developed. This may be of particular importance, from a pharmacogenetic perspective to reduce ACE2 levels in those genetically likely to have high ACE2 levels prior to infection and to increase ACE2 levels following infection with COVID-19 with genotypes associated with low ACE2 activity and/or function. Importantly, this remains a hypothesis based on data in animals and limited data in humans that deserves adequate clinical trials. Important clinical trial data would include well-designed mechanistic placebo-controlled trials of ACE2 activity and function before and following the administration of ACE-inhibitors and ARBs and large trials on the relationship between ACE2 and Ang(1–7) expression/activity on severity of infection in patients who have presented with a positive test for COVID-19. These clinical trials are needed to confirm these hypotheses and to demonstrate improvements in outcomes, while minimizing patient risk. The benefit of a trial on ACE2, COVID-19 and RAAS pharmacotherapies pharmacogenetics is that these medications are already FDA approved and most are in generic form which should shorten the time for these clinical trials while even more promising new therapies (including those that specifically target coronaviruses with a focus on membrane-bound versus circulating ACE2) are moving through phased clinical trials.

**Executive summary**

**Background**

- COVID-19 utilizes the ACE2 pathway as a means of infection.
- Early data on COVID-19 suggests heterogeneity in the severity of symptoms during transmission and infection ranging from no symptoms to death.
- The source of this heterogeneity is likely multi-faceted and may have a genetic component.
- Demographic and clinical comorbidities associated with the severity of infection suggest that possible variants known to influence the renin–angiotensin–aldosterone (RAAS) pathway (those that influence ACE2) may contribute to the heterogenous infection response to COVID-19.

**RAAS pathway & ACE2**

- ACE2 is a relatively newly discovered enzyme in the RAAS pathway and is a homolog of ACE but appears to be counteractive in nature to the function and activities of ACE and the product of ACE, Ang-II.
- ACE2 hydrolyzes Ang-I to make Ang(1–9) and Ang-II to make Ang(1–7) which then binds to a GPCR MAS.
- ACE, ACE2, Ang-II and AT1 receptor levels are high in the lung, which seems to be the primary site of complications from the corona-type viruses.
- Functional variants of renin, angiotensinogen, ACE and the AT1 receptor have been studied in detail and common variants have been shown to alter plasma levels, activity and the response to pharmacotherapy.
- Several common and functional genotypes of ACE2 have been described.
- While it is likely that a portion of the differences in COVID-19 infection rates and severity are influenced by environment or traditional demographic variables known to influence ACE2 levels, the known heterogeneity of this infection may also have genetic associations.

**Evidence that ACE2 may be associated with COVID-19 infection**

- The early data from patients who have higher rates of infection, and who have had more severe COVID-19 suggest that there are two primary common comorbidities: hypertension and diabetes.
- There is an increase in ACE2 activity in patients with hypertension.
- In addition to hypertension, ACE2 expression and activity have been shown to be elevated in animal models of both Type 1 and Type 2 diabetes.
- Along with hypertension and diabetes being common comorbidities for COVID-19, older individuals and males are more susceptible to the infection (and have demonstrated more severe cardiopulmonary complications from coronavirus), both of which are associated with differences in ACE2 expression and activity.
- Age influences ACE2 expression (mRNA and protein) in animal models and sex differences in sheep do not emerge until post puberty.

**Importance of ACE2 and Ang(1–7) augmentations post diagnosis with coronavirus respiratory infection**

- Interestingly, there is strong evidence that decreased ACE2 and Ang(1–7) levels are associated with poor prognosis in patients with respiratory dysfunction, including acute lung injury (ALI) and acute respiratory distress syndrome (ARDS). The ACE2/Ang(1–7) relationship with ACE/Ang-II is complex in that ACE2, and particularly Ang(1–7), have been shown to be primarily protective of the heart and lungs while ACE, and particularly Ang-II result in cardiopulmonary dysfunction.
- Lungs
  ○ Genetic knock-out of AT1a receptor expression markedly improved lung function in Agtr1a^-/-^ mice, confirming the function of Ang-II in lung health.
  ○ Additional work has shown that pharmacologic inhibition of the AT1 receptor attenuates the severity of acid-induced lung injury in ACE2 knockout mice and the loss of ACE2 expression in ALI leads to leaky pulmonary blood vessels through AT1a receptor stimulation.
  ○ ACE2 knockout mice developed more severe ALI in models that result in respiratory distress and animals that have more lung elastase, when compared with wild-type mice, and this loss of ACE2 results in more pronounced deoxygenation, lung edema and increased inflammatory cell infiltration.
- The heart
  ○ Previous work in coronaviruses has demonstrated that a high percentage of patients with complications have cardiac dysfunction, including cardiomyopathy and cardiac injury.
  ○ The disruption of ACE2 accelerates cardiac hypertrophy and shortens the transition period to heart failure in an Ang-II model of heart failure.
  ○ In addition, the pharmacologic inhibition of ACE2 exacerbates cardiac hypertrophy and circulating Ang(1–7) levels in transgenic rats are associated with reduced cardiac hypertrophy and fibrosis following isoproterenol infusion.
- Therefore, it is possible that variants along the RAAS that impact ACE2 levels may impact the progression of COVID-19

**Modifications of ACE2 with medications that alter the RAAS pathway**

- Previous work has demonstrated that the administration of an ACE-inhibitor or an ARB causes an increase in Ang(1–7) levels, in some studies.
- In addition, both ACE-inhibition and ARB administration increase the activity, and expression, of ACE2, which may be differential based on variants of ACE and Ang-II that have previously been shown to alter enzyme and receptor function, respectively.
- It is, therefore, possible that ACE-inhibition and ARB usage could beneficially alter ACE2 activity/expression, post infection, particularly in groups that are genetically prone to lower ACE2 activity/expression.
- Like hypertension, not all individuals will likely have the same levels of ACE2 modulation with pharmacotherapy.
